# Optimization of pressure settings during adaptive servo-ventilation support using real-time heart rate variability assessment: initial case report

**DOI:** 10.1186/s12872-016-0455-4

**Published:** 2017-01-05

**Authors:** Teruhiko Imamura, Daisuke Nitta, Koichiro Kinugawa

**Affiliations:** 1 Department of Cardiovascular Medicine, Graduate School of Medicine, University of Tokyo, 7-3-1 Hongo, Bunkyo-ku, Tokyo, 113-8655 Japan; 2 Second Department of Internal Medicine, Toyama University, 2630 Sugitani Toyama-shi, Toyama, 930-0194 Japan

**Keywords:** Heart failure, Peak end-expiratory pressure, Non-invasive positive pressure ventilation, Case report

## Abstract

**Background:**

Adaptive servo-ventilation (ASV) therapy is a recent non-invasive positive pressure ventilation therapy that was developed for patients with heart failure (HF) refractory to optimal medical therapy. However, it is likely that ASV therapy at relatively higher pressure setting worsens some of the patients’ prognosis compared with optimal medical therapy. Therefore, identification of optimal pressure settings of ASV therapy is warranted.

**Case presentation:**

We present the case of a 42-year-old male with HF, which was caused by dilated cardiomyopathy, who was admitted to our institution for evaluating his eligibility for heart transplantation. To identify the optimal pressure setting [peak end-expiratory pressure (PEEP) ramp test], we performed an ASV support test, during which the PEEP settings were set at levels ranging from 4 to 8 mmHg, and a heart rate variability (HRV) analysis using the MemCalc power spectral density method. Clinical parameters varied dramatically during the PEEP ramp test. Over incremental PEEP levels, pulmonary capillary wedge pressure, cardiac index and high-frequency level (reflecting parasympathetic activity) decreased; however, the low-frequency level increased along with increase in plasma noradrenaline concentrations.

**Conclusions:**

An inappropriately high PEEP setting may stimulate sympathetic nerve activity accompanied by decreased cardiac output. This was the first report on the PEEP ramp test during ASV therapy. Further research is warranted to determine whether use of optimal pressure settings using HRV analyses may improve the long-term prognosis of such patients.

## Background

As a non-invasive positive pressure ventilation therapy, adaptive servo-ventilation (ASV) therapy was recently developed for treating patients with heart failure that was refractory to optimal medical therapy [1]. This therapy could decrease preload and afterload, ameliorate impaired sympathetic nerve activity and increase cardiac output, eventually facilitating left ventricular reverse remodeling and improving prognosis during short- or mid-term study periods [2–8]. ASV therapy was originally developed for treating sleep-disordered breathing (SDB) but is currently being adopted for treating decompensated heart failure, irrespective of the existence of SDB [9].

Recently, Cowie et al published a prospective randomized study and indicated that ASV therapy unexpectedly decreased the survival rate compared with optimal medical therapy for patients with heart failure and SDB [10]. In this study, they utilised ASV support at relatively higher pressure settings to treat SDB. Therefore, the clinical efficacy of routine ASV therapy in all patients with heart failure has been controversial. Considering that most of other ASV studies were conducted at the default pressure setting, the optimal pressure settings may be vital to attaining improved patient prognosis with ASV therapy. Here we present the case of a patient with heart failure who was subjected to ASV support testing; we attempted to utilise several pressure settings and performed multiple haemodynamic tests, including heart rate variability (HRV) analysis and measurement of plasma catecholamine levels. To our knowledge, this is the first report on investigation of optimal pressure settings during ASV therapy.

## Case presentation

**Fig. 1:**
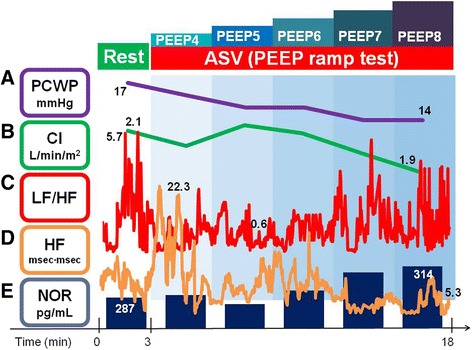
Time course of haemodynamics and autonomic nerve activity during the PEEP ramp test. PEEP, peak end-expiratory pressure; ASV, adaptive servo-ventilation; PCWP, pulmonary capillary wedge pressure; CI, cardiac index; LF, low-frequency; HF, high-frequency; NOR, noradrenalin

A 42-year-old man with normal sinus rhythm was admitted to our institution for the evaluation of his eligibility for heart transplantation owing to advanced heart failure , which was classified as New York Heart Association functional class IV due to dilated cardiomyopathy diagnosed by previous endomyocardial biopsy. He had been receiving 10 mg/d of carvedilol, 2.5 mg/d of enalapril and 25 mg/d of spironolactone. At admission, he had slight edema in bilateral leg, and his plasma B-type natriuretic peptide level was 423 pg/mL. He had no history of hypertension or diabetes at admission. Transthoracic echocardiography showed that the left ventricular diastolic dimeter was 67 mm, and as calculated by the modified Simpson method, the left ventricular ejection fraction was 23%; mild mitral and tricuspid regurgitations were present as well. He had no SDB or active respiratory infection. For further improvement of his heart failure symptoms, he was subjected to ASV support testing accompanied by haemodynamic assessment using right-heart catheterization as well as HRV and noradrenaline plasma level analyses at 10 days after the admission (Fig. 1). An informed consent was obtained from the patient beforehand.

An advanced bi-level positive airway pressure unit (AutoSet-CS; ResMed, Sydney, Australia) together with a best-fitting full-face mask (ResMed) was used for the ASV support testing, as previously described [8]. Before the final test, the patient received 5-min’s adaptation period, during which ASV support at a default setting was conducted for adaptation and confirmation of his tolerability. During the ASV support testing, the peak end-expiratory pressure (PEEP) was set at 4, 5, 6, 7 and 8 cmH_2_O, each one of which was within the minimum range of 3–10 cmH_2_O (PEEP ramp test); each pressure setting was maintained for 3 min.

During the PEEP ramp test, the pulmonary capillary wedge pressure and cardiac index gradually decreased with the increased PEEP settings, although a maximal cardiac index was achieved at a PEEP of 5 cmH_2_O (Fig. 1a and b). Other haemodynamic parameters, including heart rate and systolic blood pressure, remained unchanged from the baseline levels of 84 bpm and 110 mmHg, respectively, during the study period.

During the PEEP ramp test, we also performed an HRV analysis (Fig. 1c and d). Power spectral analyses of HRV were performed using the MemCalc power spectral density method (MemCalc system; Suwa Trust Co, Tokyo, Japan) [11–14]. Low frequency (LF) was defined as a level of 0.04–0.15 Hz; high frequency (HF), which reflected parasympathetic nerve activity, was defined as a level of 0.15–0.40 Hz. The ratio of LF to HF (LF/HF ratio) reflected sympathetic nerve activity. Although the LF/HF ratio decreased upon the initiation of ASV support, the LF/HF ratio gradually increased at increased PEEP levels (Fig. 1c) accompanied by increased noradrenaline plasma levels (Fig. 1e). In contrast, the HF level gradually decreased below the baseline at increased PEEP levels (Fig. 1d). The patient complained of mild discomfort at a PEEP of 8 cmH_2_O. No adverse events occurred during the study period.

## Discussion

### Optimization of ASV therapy

The clinical efficacy of ASV therapy in all patients with heart failure has been controversial. Although previous studies have shown the efficacy of ASV therapy in ameliorating symptomatic congestion, facilitating left ventricular reverse remodeling and improving survival rate of patients with mild-to-advanced heart failure [3–8], recent studies have not been able to show any definitive advantage of ASV therapy over optimal medical therapy [2, 10]. Considering these results, optimal patient selection and optimal pressure settings may be key to successful ASV therapy.

Momomura et al performed a prospective randomized study [2], which did not demonstrate the significant advantages of ASV therapy compared with optimal medical therapy. Considering that the enrolled patients were relatively less sick, the patients who were not receiving optimal medical therapy may not be good candidates for ASV therapy. Consistently, Yamada et al demonstrated that cardiac output increased more in patients with higher pulmonary capillary wedge pressures and mitral regurgitation during ASV therapy [15]. Decreases in preload and afterload during ASV therapy may more effectively reduce intracardiac pressure in patients with advanced heart failure than in those with mild heart failure . In contrast, we recently demonstrated that patients with a severely remodeled left ventricle with long-term heart failure rarely responded to ASV therapy [16]. Considering this evidence, further studies are warranted to identify suitable patients for ASV therapy.

### Changes in clinical variables during PEEP ramp test

Cowei et al administered ASV therapy, probably for treating SDB, at relatively higher pressure settings (higher than the default level), i.e., PEEP of 5 cmH_2_O, and found that ASV therapy unexpectedly increased mortality [10]. Therefore, another concern was the optimization of pressure settings during ASV therapy. Here, to our knowledge, this is the first report on the PEEP ramp testing during ASV support.

Positive pressure ventilation generally relieves elevated intracardiac pressure due to the decrease in preload and increase in cardiac output [2]. However, very high pressure can reduce cardiac output, as shown in Fig. 1b. Considering the haemodynamics of the patient, a PEEP of 5–6 cmH_2_O would be the optimal pressure setting at least in this case.

We recently showed the usefulness of real-time HRV assessment during ASV support [17]. In the same manner, we quantified the time course of autonomic nerve activity during the PEEP ramp test. The LF/HF ratio increased along with increase in PEEP (Fig. 1c). Sympathetic nerve activity may be stimulated by decreased cardiac output due to the inappropriately reduced preload [13]. The HF level decreased gradually along with increase in PEEP (Fig. 1d). Discomfort at higher PEEP, similar to that at which the present patient complained, may reduce parasympathetic nerve activity [17]. Based on the hypothesis that suppression of sympathetic nerve activity and stimulation of parasympathetic nerve activity result in a favorable prognostic effect in patients with heart failure [13, 18], a PEEP of 5–6 cmH_2_O may also be the optimal pressure setting at least in this case.

### Future directions

The validity of this PEEP ramp test should be confirmed in many patients. Considering our previous study results [13], we attempted each PEEP setting for 3 min; however, the optimal period also should be confirmed in a large-scale population study. We did not measure the trend for the respiratory pattern including the respiratory rate and variations in tidal volume, which may affect sympathetic nerve activity [6]. Such respiratory data also would strengthen the importance of the PEEP ramp test. Also, we did not measure blood gases at each pressure setting considering its invasiveness. Haemodynamics variables are subject to the pH values and blood gases, and these data may strengthen the interpretation of the PEEP ramp test. Here, we sought the optimal pressure settings for haemodynamics and autonomic nerve activity during short-term ASV support in a patient. Although responses to long-term ASV therapy remain unclear, the optimal pressure setting accompanied by sufficiently preserved cardiac output and normalized autonomic nerve activity may improve patient prognosis. The optimal pressure setting may vary for each patient, depending on age, co-morbidity, presence of SDB and degree of heart failure . ASV therapy at the optimal pressure setting for each patient, as determined by the PEEP ramp test, may improve patient prognosis compared with ASV therapy performed at the default pressure setting. A prospective randomised trial of the PEEP ramp test is warranted.

## Conclusions

We performed here for the first time a PEEP ramp test during ASV therapy. Whether optimization of PEEP setting using HRV analyses may improve the long-term prognosis should be confirmed in the prospective study.

## Abbreviations

ASV: Adaptive seroventilation
HF: High frequency
HRV: Heart rate variability
LF: Low frequency
LF/HF ratio: Ratio of LF to HF
PEEP: Peak end-expiratory pressure
SDB: Sleep-disordered breathing
